# Fracture panfaciale: un challenge thérapeutique: à propos d'une observation et revue de la littérature

**DOI:** 10.11604/pamj.2015.20.149.1501

**Published:** 2015-02-17

**Authors:** Malika Fassih, Hicham Nassik, Mourad Nafaa Redallah Abada, Sami Rouadi, Mohamed Mahta, Mohamed Roubal, Mustapha Essaadi, Mohamed Fatmi El Kadiri

**Affiliations:** 1 Hôpital 20 Août, CHU Ibn Rochd, Casablanca, Maroc

**Keywords:** Traumatisme, fracture, face, panfaciale, trauma, fracture, face, panfaciale

## Abstract

La gestion des traumatismes de la face a subi plusieurs progrès dans la dernière décennie. Avec l'avènement de la tomodensitométrie bi- et tridimensionnelle, qui permet de dresser une cartographie précise des lésions osseuses, le développement du matériel d'ostéosynthèse, et les procédés de réduction des fractures. L'objectif est la restauration totale de la forme et la fonction de la face, seul garant pour empêcher la survenue de préjudices esthétiques et fonctionnels. Nous rapportons l'observation de Mme assal khadija, âgée de 38 ans, admise le 25 Aout 2010, au service des urgences d'ORL et maxillo-faciales, à l'hôpital 20 aout de Casablanca, victime d'un AVP, occasionnant un traumatisme facial grave, avec un important délabrement de la face, et asphyxie. Une trachéotomie a été réalisée en urgence. Le bilan lésionnel a objectivé un important fracas du massif facial intéressant l’étage supérieure, moyen et inférieur. Dans l'immédiat, un parage a été réalisé sous anesthésie générale, la réparation des parties molles délabrées a été effectuée en endobuccale (vestibules, langue et plancher buccal) et pour les téguments de la face. Une semaine après, la patiente a été reprise pour la reconstruction du massif facial, nous avons opté pour le «inside-out, de bas en haut" pour la réduction des fractures. Nous avons procédé à la fixation de la mandibule qui a précédé la reconstruction du maxillaire, de la pyramide nasale, et de l'orbite. L’évolution a été marquée par l'apparition d'un orostome, qui fera l'objet après tarissement de l'infection, d'une réparation grâce à un lambeau myocutané, et une nécrose cutanée de la région zygomatique laissée à la cicatrisation dirigée. Toutes les lésions traumatiques de la face ont des répercussions esthétiques et fonctionnelles à moyen et long termes, dont le degré dépend de la qualité de la prise en charge, qui étant la plus précoce et la plus complète possible avant la consolidation des fractures est le meilleur garant d'un résultat satisfaisant à long terme.

## Introduction

La gestion des traumatismes complexe de la face a subi plusieurs changements dans la dernière décennie. La prise en charge a bénéficié des progrès de l'imagerie, des procédés chirurgicaux et du matériel d'ostéosynthèse, qui ont modifié le pronostic, diminué le temps d'intervention et de cicatrisation, diminué aussi le nombre et l'importance des séquelles.

Les traumatismes complexes de la face constituent un véritable challenge thérapeutique, pour la restitution complète de la forme et de la fonction de la face. Dans notre pays, les accidents de la voie publique demeure la principale cause. Le traitement chirurgical est une urgence qui peut être différée dans certaine situations, cependant les vraies urgences sont représentées par l'asphyxie, l'hémorragie, et les lésions intracrâniennes associées.

## Patient et observation

**Figure 1:**
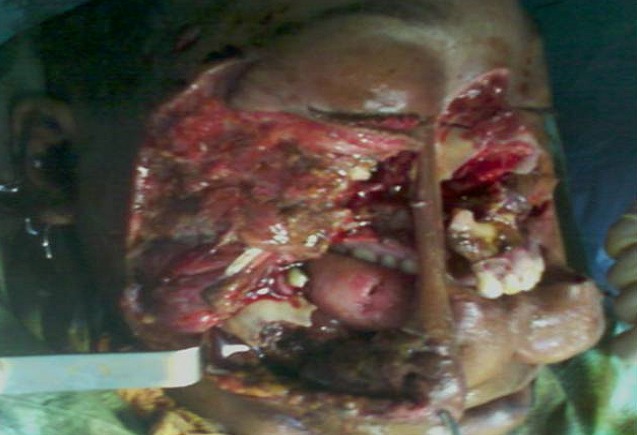
Délabrement de la face avec impaction du maxillaire droit

**Figure 2:**
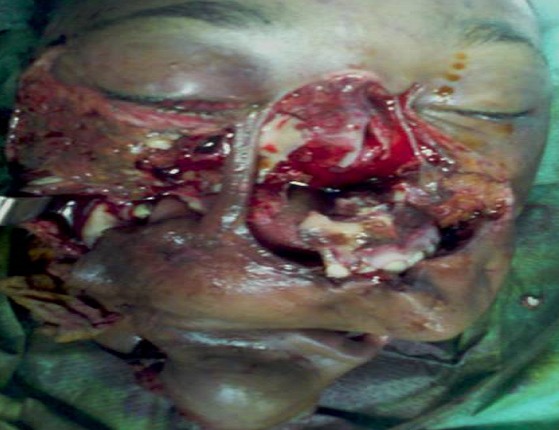
Détachement de l'hémi maxillaire gauche avec fracture comminutive des OPN

**Figure 3:**
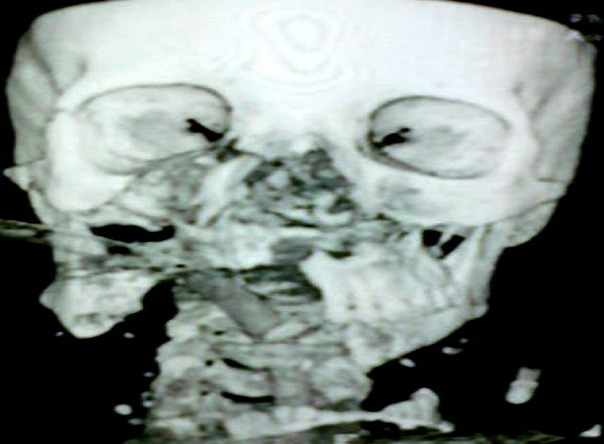
TDM de la face 3D: fracture comminutive du maxillaire et des OPN. Déplacement du fragment symphysaire détaché en bas avec les parties molles

Inclure votre observation clinique ici. Si plusieurs patients, utilise le pluriel, Patients et observations. Mme A.k, âgée de 38 ans, mariée, femme au foyer, résidente en Italie. Admise le 25Aout 2010, au service des urgences d'ORL et maxillo-faciales, à l'hôpital 20 Aout de Casablanca, victime d'un AVP, occasionnant un traumatisme facial grave, avec un délabrement important de la face et asphyxie ayant nécessitée une intubation orotrachéale difficile en urgence. La patiente présentait une altération de la conscience avec un score de 13/15 sur l’échelle de Glasgow. Après stabilisation, une TDM crânio-faciale avec des reconstruction 3D a été réalisée. La TDM cérébrale s'est révélée normale. La patiente fut adressée aux urgences ORL et maxillofaciale pour prise en charge. La malade est arrivée chez nous en arrêt respiratoire, certainement due à l'extubation au cours du transfert. Une trachéotomie salvatrice a été réalisée en urgence, devant l'impossibilité de la ré intuber. L'examen physique de la patiente objective un important délabrement des parties molles de la face épargnant seulement la région frontale et palpébrale supérieure (Figure 1, Figure 2, Figure 3). Ouverture des 2 vestibules, et du plancher buccal intéressant les muscles et la muqueuse, désinsertion de la langue et plusieures plaies de la face dorsale. Un Fracas facial qui a inclus: une fracture comminutive du maxillaire, des os propres du nez, du rebord orbitaire inférieure droit, une fracture bifocale de la mandibule avec segment symphysaire mobile occasionnant une glossoptose à l'origine de l'asphyxie. Il y avait des pertes dentaires multiples sur l'arcade inférieure. L'examen ophtalmologique a objectivé un éphéma de l’œil droit.

**Figure 4:**
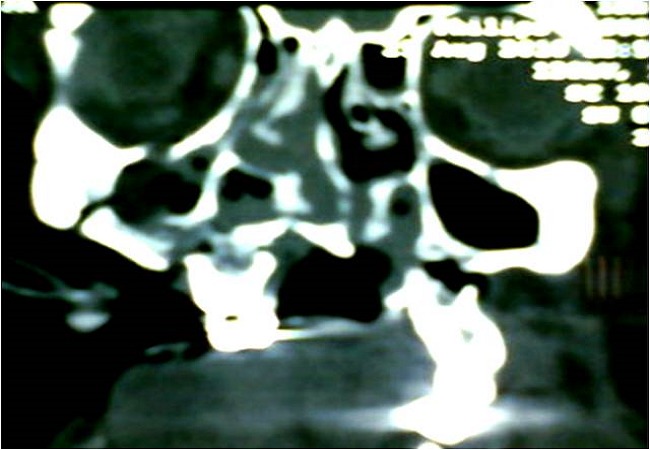
TDM face coupe coronale fracture 2 apophyses ptérygoïdes et fracture du plancher orbitaire droit

**Figure 5:**
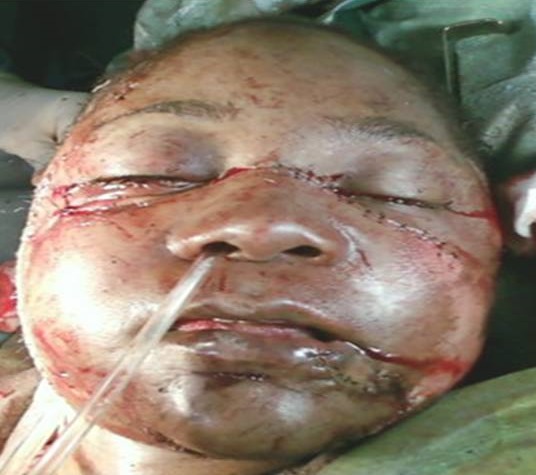
Résultat post opératoire immédiat après reconstruction

La TDM faciale avec reconstruction 3D a montré une fracture Le fort I à gauche, un Le fort III à droite avec impaction de l'hémi maxillaire Dt, fracture des 2 apophyses ptérygoïdes, fracture comminutive des os propres du nez, fracture parasymphysaire bifocale de la mandibule détachant la région symphysaire déplacée en bas avec les parties molles, et fracture du plancher de l'orbite droit (Figure 4, Figure 5). La TDM du rachis cervical s'est révélée normale ***Dans l'immédiat*** et sous anesthésie générale, après trachéotomie, le traitement a consisté en un parage laborieux: décontamination, extraction des corps étrangers (dents, fragments d'os), excisions des tissus dévitalisés, régularisation des berges des lambeaux. Dans un 2^ème^ temps, une réduction immobilisation des 2 arcades maxillaire et mandibulaire avec mise en place de 2 arcs dentaires a été effectuée. Les sutures ont été réalisées plan par plan sans tension. Les suture des bords du lambeau cutanéo-muqueux au niveau des paupières inférieures était soigneuse sans tension, avec reconstruction des canthus. La muqueuse endobuccale a été suturée, d'abord en réparant les deux vestibules, le plancher buccal, et amarrage de la langue au plancher. La vérification de l'intégrité du sténon s'est révélée normale. La pyramide nasale fut ensuite réamarrée au pourtour de l'orifice piriforme et la columelle fixée à l’épine nasale antérieure sur la ligne médiane, tout en s'assurant de la symétrie des narines. La perméabilité des deux fosses nasales a été vérifiée avec mise en place d'un tamponnement antérieure. la deuxième intervention a été programmée *1 semaine après*, jusqu’à fonte des œdèmes, elle a consisté en la reconstruction du massif facial selon le «inside-out, de bas en haut": durant le 1^er^ temps: une réduction-fixation des fractures mandibulaires a été réalisée à l'aide de 4 miniplaques à quatre trous, après obtention d'un bon articulé stabilisé par un blocage intermaxillaire.

Le 2^ème^ temps a permis la reconstruction de l’étage moyen de la face: réduction et fixation rigide des fractures Lefort grâce à des microplaques, et restauration de l'orifice piriforme. Le rebord orbitaire inférieure a été réparé et fixé à l'aide d'une microplaque, et du fil d'acier. Il n y avait pas d'incarcération musculaire. En fin, une réduction des Os Propres du nez a été réalisée avec mise en place d'un plâtre. L'Evolution a été marquée par la survenue d'un orostome qui a été traité par un lambeau du grand pectoral avec un bon résultat. On a assisté à une nécrose cutanée de la région zygomatique droite qui a té laissée à la cicatrisation dirigée.

## Discussion

Les traumatismes complexes de la face constituent un véritable challenge thérapeutique, pour la restitution complète de la forme et de la fonction de la face. Les étiologies aux pays du Maghreb sont représentées par les AVP, les rixes, et les accidents de travail. Aux USA, ce sont les agressions par tires de balle, les AVP et les accidents de sport qui sont les plus fréquentes [1]. Quelques soient les séries, le sexe masculin est le plus touché [2]. La traumatologie faciale fait partie des urgences chirurgicales. Les vraies urgences vitales sont cependant peu nombreuses. L'obstruction des voies aériennes est la principale cause de décès rapide d'un traumatisé facial. Assurer la liberté des voies aériennes supérieures est donc une urgence vitale. Ceci tout en protégeant le rachis cervical. Des obstructions majeures des voies aériennes supérieures se rencontrent dans les fractures complexes du maxillaire supérieure, en particulier dans les fractures de Lefort avec enfoncement et déplacement importants, ou de la mandibule notamment dans les fractures bifocales avec glossoptose. Les autres causes d'asphyxie sont plus classiques: elles sont dues à la présence de sang et de vomissements dans l'oropharynx, aux chutes des dents avulsés ou de fragments de prothèse dans la filière respiratoire chez un patient en coma, et à l’œdème traumatique des parties molles oropharyngées [3, 4].

L'abus d'alcool, fréquemment associé à ces traumatismes, ainsi que les traumatismes crâniens associés, par altération de la conscience, altèrent la ventilation par abolition des reflexes protecteurs des voies aériennes supérieures [5]. La sécurité des voies aériennes peut généralement être obtenue par intubation orotrachéale, qui est réussie dans plus de 83% [6]. Cependant, elle peut s'avérer difficile voire impossible en raison des fractures et des délabrements faciaux modifiants l'anatomie normale, à l’œdème des tissus mous, au saignement, et aux lésions associées du rachis cervical. Certains auteurs ont rapporté l'utilisation du nasofibroscope dans les intubations difficiles [7]. Si les tentatives d'intubation échouent, une cricothyroïdotomie, ou une trachéotomie urgente est le seul moyen salvateur. Dans la littérature, une voie aérienne urgente est nécessaire dans 35% des patients présentant ces lésions [8]. Notre patiente a nécessité la réalisation d'une trachéotomie salvatrice en raison d'un délabrement maxillo-facial spectaculaire entrainant une modification de l'anatomie normale rendant l'intubation orotrachéales impossible, la patiente a été reçue en détresse respiratoire avec trouble de conscience et chute de la langue.

Il y a une forte association entre les traumatismes complexes de la face et les traumatismes crâniens, pouvant occasionner des contusions cérébrales, des hémorragies intracrâniennes, ou des fractures-embarrures. Par conséquent, tout traumatisé de la face doit bénéficier d'un examen neurologique minutieux, et au moindre doute sur une lésion cérébrale, une TDM crânio-cervicale doit être demandée. Demetriades [6] a rapporté une incidence de 17% des lésions cérébrales associées, les lésions du rachis cervical ont été trouvé dans 8% des cas. Les autres urgences vitales sont représentées par les hémorragies massives suite aux plaies faciales avec lésion de l'artère faciale ou temporale, le contrôle est obtenu par compression externe, ou ligature vasculaire. Des épistaxis secondaires à des fractures du tiers moyen de la face peuvent nécessiter un tamponnement antérieur voire postérieure selon l'importance de l'hémorragie. L'hémostase peut être assurée par une sonde de foley. Une hémorragie buccopharyngée incoercible peut aussi faire l'objet d'un tamponnement orobuccale après trachéotomie.

La TDM de la face en coupes millimétriques, avec des coupes coronales, sagittale, et axiales, permet de dresser une cartographie des lésions osseuses: le nombre de fractures, les déplacements, établir une classification qui guide les indications chirurgicales et les procédés thérapeutiques. Les reconstructions en 3D constituent l'un des progrès de l'imagerie, devenue actuellement indispensable avant la décision thérapeutique. Concernant les fractures panfaciales, il n'existe pas de classification claire et unanime dans la littérature. La définition la plus connue et la plus utilisée est: l'existence de fractures simultanées au niveau des 3 étages de la face: le tiers inférieur mandibulaire, le tiers moyen maxillo-naso-zygomatique et le tiers supérieur orbito-frontal [9].

L'association de fracture de la mandibule, du maxillaire et du complexe zygomatique: constitue une définition récente. Les auteurs précisent qu'elle est fréquemment accompagnée d'une fracture du CNEMFO: complexe naso- ethmoido-fronto- orbitaire [10]. Les fractures du maxillaire sont classées en fonction d'un système proposé par Le Fort, en 1901. Il ya trois types, Le Fort I, II ou III, et ils sont déterminés par le type et la localisation des traits de fractures. Les Fractures de la mandibule sont également classées selon la localisation anatomique. La nécessité et la nature d'une intervention chirurgicale sont déterminées par le type et l'emplacement de la fracture. Le but du traitement est de restaurer la forme et la fonction de la face, tout en évitant les séquelles inesthétique et les répercussions fonctionnelles. Plusieurs procédés chirurgicaux: bottom to top, “top to bottom”, “inside-out” ou “outside-in” ont été décrits dans la littérature [10]. Le «inside-out, de bas en haut” était le principe directeur le plus utilisé dans la gestion des traumatismes panfaciaux. La fixation de la mandibule dentaire guide la reconstruction de l’étage médio-facial pour la restauration d'un bon articulé. En effet, beaucoup de chirurgiens préfèrent la mandibule comme une base sur laquelle reconstruire l'articulé dentaire. Une mandibule correctement reconstruite rétablira la largeur inférieure et la hauteur postérieure de la face.

Avec l'avènement de la fixation rigide (ostéosynthèse), la reconstruction médio-faciale peut précéder la fixation de la mandibule, si les piliers sont bien restaurés [11]. En effet pour les cas où le maxillaire et la mandibule sont fracturés simultanément, interrompant les 2 arcades dentaires, il est difficile de rétablir un articulé dentaire correct, Kelly [12] a suggéré de réduire et de stabiliser le palais dur d'abord comme un guide pour la reconstruction de la mandibule.

Gruss et Phillips [13] conseillent la réduction de l'arcade zygomatique et la projection du ceintre malaire comme une première étape dans le traitement, pour rétablir la largeur supérieure de la face avant la reconstruction du CNEMFO, du maxillaire et de la mandibule. Merville [13] atteste que la réduction des fractures devrait procéder de «haut vers le bas“ si la région ethmoido-naso-orbitaire est touchée.

L'utilisation de greffe osseuse est recommandée s'il ya une perte osseuse significative ou des fracture hautement comminutives. Dans certaines circonstances, l'utilisation de blocage intermaxillaire-donne une stabilisation suffisante des fractures, sans fixation interne supplémentaire. Il est utilisé dans les reconstructions immédiates, lorsque les tissus de couverture sont atteints. Cependant, le plus souvent, les fractures maxillo-faciales sont stabilisés, en utilisant des matériaux d'ostéosynthèse par mini plaque. Il ya une controverse importante au sujet du timing de la chirurgie des fractures panfaciales.

Il ya de nombreux avantages à un traitement précoce, non seulement il permet de réduire les risques infectieux postopératoires, mais empêche également la rétraction des parties molles superficielles. Malheureusement, les patients qui sont instables à cause des traumatismes associées neurologiques ou systémiques, la réparation d'une fracture faciale peut être retardée jusqu’à stabilisation de ces lésions. Un retard de 3 semaines pour la réparation définitive, augmente la difficulté d'obtenir une réduction anatomique parfaite des fractures déplacées. En effet, les bords fracturaires commencent à se résorber, cela peut conduire à un cal vicieux, un retard de consolidation, ou à une pseudarthrose [10].

Par ailleurs, beaucoup de chirurgiens aujourd'hui ont tendance à retarder l'intervention chirurgicale jusqu’à résorption des ‘dèmes, et que la symétrie de la face puisse être mieux appréciée [14]. Les dents délabrées ou non viables dans le trait de fracture doivent être enlevés car ils peuvent favoriser l'infection et le sepsis [15]. Une attention particulière doit être donné à la réparation des lésions des parties molles superficielles, notamment, les plaies péri-orificielles qui doivent être soigneusement suturées afin d’éviter les séquelles aussi bien esthétiques que fonctionnelles. A l'heure actuelle, une réparation définitive immédiate, après décontamination soigneuses et débridement des plaies, est préconisée par de nombreux auteurs. Les lésions muqueuses sont fermées sans tension lorsque cela est possible. En cas de vastes pertes de substance, la muqueuse est fixée à la peau pour éviter les rétractions. L'utilisation de lambeaux d'avancement ou de rotation peut être réalisée [16]. Les patients présentant des lésions des paupières doivent bénéficier d'un examen ophtalmologique soigneux pour détecter les lésions oculaires et celles des voies lacrymales. La présence d’énophtalmie ou d'une exophtalmie doit être reconnue, et faire rechercher une fracture du cadre orbitaire notamment du plancher. L'association avec une diplopie fait suspecter une incarcération du muscle grand oblique, ce qui constitue une indication chirurgicale urgente. La paralysie faciale est une autre urgence fonctionnelle, dont le diagnostic peut être rendu difficile, en cas d’‘dèmes et de délabrements importants, ou chez un patient en coma. Le diagnostic de section nerveuse impose une réparation dès que possible, idéalement dans les 72 heures. Une lésion du canal de sténon est suspectée devant une plaie sur la ligne tragus- commissure labiale. La vérification de l'intégrité du canal est effectuée par cathétérisme à partir de l'ostium jugale. Un calibrage est indiqué en cas de rupture, afin d’éviter les fistules et les kystes salivaires. L'infection est un autre souci du chirurgien, représentée essentiellement par l'ostéite et l'infection des parties molles. La dissémination est expliquée par la communication des foyers contus et fracturaires avec des cavités septiques telles que la cavité orale, nasale et les sinus. Se basant sur ces données, l'utilisation d'antibiotiques est recommandée.

## Conclusion

Malgré les progrès réalisés en matière de prise en charge des traumatisés faciaux, la survenue de répercussions esthétiques et fonctionnelles est encore fréquente. Il s'agit de séquelles essentiellement esthétiques dues aux cicatrices disgracieuses, des asymétries faciales, des déformations de la pyramide nasale. Des troubles de l'articulé dentaire sont fréquentes. Les séquelles ophtalmologiques peuvent être à type d’énophtalmie et de dystopie canthale. Les séquelles neurologiques sont représentées par la paralysie faciale et certaines anesthésies trigéminales. Ces séquelles sont souvent associées à un retentissement psychologique important vue le rôle primordial de l'image de soi dans les relations sociales, ce qui exige un accompagnement psychologique de ces malades durant toute la période du traitement pouvant nécessiter des interventions multiples.

## References

[1] Shuker ST (2010). Maxillofacial air-containing cavities, blast implosion injuries, and management. J Oral Maxillofac Surg..

[2] Bellavoir A, Suleau J, Jouen F, Pons J (1984). Considérations statistiques à propos des fractures sinusales de la face. Rev Stomatol Chir Maxillo-fac..

[3] Ardekian L, Rosen D, Klein Y, Peled M, Michaelson M, Laufer D (1998). Life-threatening complications and irreversible damage following maxillofacial trauma. Injury..

[4] King HK (1996). Airway managements of patients with maxillofacial trauma. Acta Anaesthesiol Sin..

[5] Perry M, Morris C (2008). Maxillofacial injuries and airway management dilemmas. International Journal of Oral and Maxillofacial Surgery..

[6] Demetriades D, Chahiuan S, Gomez H (1998). Initial evaluation and management of gunshot wounds to the face. J Trauma-Injury Infection Critical Care..

[7] Feliciano DV, Feliciano DV, Moore EE, Mattox KL, CT (2003). Patterns of injury. Trauma.

[8] Dolin J, Scalea T, Mannor L, Sclafani S, Trooskin S (1992). The management of gunshot wounds to the face. J Trauma..

[9] Christophe MU TRAUMATOLOGIE DE LA FACE, Faculté de Médecine Strasbourg - DCEM1 2004/ 2005 - Module 12B - Appareil Loco-Moteur. http://www-ulpmed.u-strasbg.fr/medecine/cours_en_ligne/e_cours/pdf-locomoteur/05_traumatologie_de_la_face.pdf.

[10] Dongmei He, Yi Zhang, Edward Ellis (2007). Panfacial Fractures: Analysis of 33 Cases Treated Late. Dongmei Journal of Oral and Maxillofacial Surgery..

[11] Wenig BL (1991). Management of panfacial fractures. Otolaryngol Clin North Am..

[12] Kelly KJ, Manson PN, Vander Kolk CA (1990). Sequencing LeFort fracture treatment (Organization of treatment for a panfacial fracture). J Craniofac Surg..

[13] Gruss JS, Phillips JH (1989). Complex facial trauma: The evolving role of rigid fixation and immediate bone graft reconstruction. Clin Plast Surg..

[14] Cummings CW (2005). Otolaryngology: Head & Neck Surgery.

[15] Kihtir T, Ivatury R, Simon R, Nassoura Z, Leban S (1993). Early management of civilian gunshot wounds to the face. J Trauma..

[16] Motamedi MH (2003). Primary management of maxillofacial hard and soft tissue gunshot and shrapnel injuries. J Oral Maxillofac Surg..

